# Antioxidant activity of various Mauritanian date palm (*Phoenix dactylifera* L.) fruits at two edible ripening stages

**DOI:** 10.1002/fsn3.167

**Published:** 2014-09-01

**Authors:** Fouteye Mint Mohamed Lemine, Mohamed Vall O Mohamed Ahmed, Lobna Ben Mohamed Maoulainine, Zein el Abidine O Bouna, Abdoulaye Samb, Ali O Mohamed Salem O Boukhary

**Affiliations:** 1 UR-Génomes et milieux, Faculté des Sciences et Techniques, Université des sciences, de technologie et de médecineBP 5026, Nouakchott, Mauritania; 2 Laboratoire des produits naturels, Faculté des Sciences et Techniques, Université Cheikh Anta DiopDakar, Senegal; 3 Département de production et protection végétale, Institut Supérieur de l'Enseignement TechnologiqueRosso, Mauritania

**Keywords:** Antioxidant activity, date palm fruit, flavonoids, Mauritania, phenolics, *Phoenix dactylifera*, ripening

## Abstract

The increasing interest in plant phenolics and flavonoids outlined the necessity of determining their contents and biological activity in Mauritanian date palm fruits. Methanolic extracts of fruit of six date palm cultivars commonly grown in Mauritania were screened for their antioxidant activity, total phenolics, and flavonoid content at two edible ripening stages. Polyphenols and flavonoids were higher in the*Blah* stage, corresponding to*Khalal* in the standard Iraqi Arabic nomenclature, compared to the fully mature*Tamr* stage regardless the cultivar. The average of total phenolics at*Blah* and*Tamr* stages were 728.5 and 558.9 mg gallic acid equivalents (GAE) per 100 g dry matter (DM), whereas the average flavonoid content was 119.6 and 67.3 mg quercetin equivalents (QE) per 100 g DM, respectively. The*Blah* stage also exhibited the highest total antioxidant activity with a Trolox equivalents antioxidant capacity (TEAC) value of 129.3 *μ*mol/100 g DM founded in Bou seker's Blah date, followed by Tijib cultivar with TEAC value of 114.3 *μ*mol/100 g DM and an average TEAC value of 107.5 *μ*mol/100 g DM. Furthermore, a high positive correlation was found between total phenolics in*Tamr* (*r* = 0.92) and*Blah* (*r* = 0.87) stages and TEAC of fruit methanolic extracts compared to the flavonoids, suggesting that phenolics were the major contributor to the antioxidant activity.

## Introduction

The date palm tree,*Phoenix dactylifera* L., is the most important subsistence crop in Mauritania and all of North Africa. At present, more than 5000 cultivars of date palm are known to exist all over the world (Ibrahim 2008), but only a few important ones have been evaluated for their agronomic performance and fruit quality. Date palm fruit is composed of a fleshy pericarp, a membranous endocarp and seed. The main ripening stages of dates are known worldwide by their Iraqi Arabic names as*Kimri* (unripe),*Khalal* (full-size, slightly cruchy, edible),*Rutab* (ripe, soft, edible) and*Tamr* (ripe, reduced moisture; edible) (Ahmed et al. 1995). Dates are highly nutritious because of its rich sugar content in the form of fructose, glucose, and sucrose (Reynes et al. 1994; El-Shibli and Korelainen 2009), and as a good source of dietary fiber (Al-Shahib and Marshall 2002; Mrabet et al. 2012) and essential minerals and vitamins (Yousif et al. 1982; Chaira et al. 2009). In addition, dates have been reported to contain a variety of polyphenols, including phenolic acids, hydroxycinnamates, flavonoid glycosides, and proanthocyanidins (Al-Farsi et al. 2005; Mansouri et al. 2005; Hong et al. 2006) which make it an interesting fruit from a pharmaceutical and medicinal point of view. Mauritania has 2.6 million date palm trees producing some 58,000 tons of dates annually and representing a source of income for 16% of the dwellers living in the date-producing regions (Anonymous 2012). Nearly half of the date's production is freshly consumed at*Khalal* locally called*Blah* and*Rutab* or*Tamr* stages depending on the cultivar during the harvest season (June, July, and August) making it the most consumed fruit in Mauritania with an average per capita consumption of 7 kg per year (Chetto et al. 2005). Date processing technology (date packing, syrup, jam, paste, …) is still rudimentary. Some traditional uses of dates as medicine were also reported (Leriche 1952).

Studies have indicated that phenolic compounds are a major source of natural antioxidants in foods of plant origin (Hergmann et al. 1998) and exhibit a wide spectrum of biochemical activities such as antimicrobial, antimutagenic, anticarcinogenic as well as the ability to modify the gene expression (Tapiero et al. 2002; Nakamura et al. 2003). Numerous epidemiological studies confirm significant relationship between the high dietary intake of flavonoids and the reduction of cardiovascular and carcinogenic risks (Cook and Samman 1996). Various factors (climatic, agronomic, genomic, pre- and post-harvest conditions and processing) may affect the chemical composition of plant foods and may have a significant role in determining the phenolics composition and the bioactivity of these compounds (Imeh and Khokhar 2002). The ripening stage is another important factor that may influence the compositional quality of fruit and vegetables. Several investigations on phenolic compounds and their biological activities of various date palm fruits from different date-growing countries have been recently published (Chaira et al. 2009; Saleh Abdulla et al. 2011; Eid et al. 2013). Such studies on Mauritanian date palm cultivars are lacking. Therefore, the present investigation is carried out to compare total phenolics, flavonoids content, and antioxidant activity in two edible stages of fruit development of six date palm cultivars. Such a study will be of great interest in establishing a research program aiming at the valorization of local germplasm.

## Materials and Methods

### Sources of standards and solvents

Standards of Gallic acid, quercetin, 6-hydroxyl-2,5,7,8-tertramethylchrome-2-carboxylic acid (Trolox), and 1,1 diphenyl-2-picrylhydrazyl (DPPH) were from Sigma-Aldrich (Munich, Germany). All other chemical reagents used were of analytical grade.

### Plant material

Fresh date fruits were from six commonly grown cultivars originating from Atar (20°31′N; 13°03′W; altitude: 220 m) and Tijigja (18°31′N; 11°24′W; altitude: 400 m). They were collected at two edible ripening stages namely*Blah* corresponding to*Khalal* stage in the standard Iraqi nomenclature and*Tamr* (Table1). Identification of each cultivar was visually verified by the experienced farmers. The first stage of maturation was harvested in May and the last stage in July 2013. All fruits were rapidly cooled and transported to the laboratory for chemical analysis.

**Table 1:** Name, origin, fruit characteristics, and eating quality of date palm cultivar analyzed.

Cultivar	Orgin	Fruit consistancy^1^	Fruit color^2^	Eating quality	Studied maturity stages
Ahmar dli	Atar	Semi-soft	Dark red	Excellent	*Blah, Tamr*
Ahmar denga	Tijigja	Semi-soft	Dark red	Excellent	*Blah, Tamr*
Bou seker	Atar	Dry	Yellow	Medium	*Blah, Tamr*
Tenterguel	Tijigja	Semi-soft	Yellow	Medium	*Blah, Tamr*
Lemdina	Atar	Soft	Yellow	Good	*Blah, Tamr*
Tijib	Atar	Soft	Red	Good	*Blah, Tamr*

1 At the*Tamr* stage.

2 At the*Blah* stage: In Mauritania, the maturity stage locally known as “*Blah*” corresponds, in the Iraqi standard nomenclature, to the “*Khalal*” maturity stage.

### Determination of fruit DM

The*Blah* and*Tamr* stages of each date palm cultivar were pitted and 20 g of the corresponding pulps were cut into small pieces and allowed to dry in the oven with an air flow of 60°C until constant weight is reached (24H for*Blah* flesh and 72H for*Tamr* pulp depending on the fruit consistency). The percentage of DM was then determined using the formula: % DM = (dry sample weight/wet sample weight) × 100. Plant materials were then grounded with a mixer and stored in hermetic glass bottles at dark until extraction processing.

### Preparation of fruit extract

A portion of 10 g of dried date fruit material from each ripening stage was extracted with 30 mL of methanol (80%), after being mixed by a magnetic stirrer for 1 h, and then filtered with Whatman no. 2 filter paper. The homogenate was centrifuged at 8000 *g* for 30 min at 4°C. The supernatant was recovered and the pellet re-extracted three times under the same conditions. All resulting supernatants were pooled and the solvent was evaporated under vacuum. The extracts were then redissolved in 10 mL of the same solvent. These concentrated extracts were used to determine total phenolics, flavonoids content, and antioxidant activity of date palm fruits.

### Determination of total phenolics

The total phenolics was quantified for each date extract according to the method described by Singleton et al. (1999) using the Folin–Ciocalteu reagent and gallic acid as a reference standard. Aliquots of 0.2 mL of each sample were tested in triplicate, and 0.5 mL of Folin-Ciocalteu phenol reagent (0.2 mol/L) was added to each tube. The tubes were maintained at room temperature for 5 min, afterward, 0.4 mL of 7.5% sodium carbonate (Na_2_CO_3_) was added and mixed well then, the samples were incubated for 60 min at 25°C. The absorbance was measured at 750 nm with a UV/visible spectrophotometer. Results were expressed as milligram gallic acid equivalents (GAE)/100 g DM. The standard curve was prepared with gallic acid in five different concentrations (10, 25, 50, 100, and 200 mg/L).

### Determination of flavonoids content

The content of flavonoids was measured spectrophotometrically using the aluminum chloride colorimetric method (Chang et al. 2002) and expressed as milligram quercetin equivalents (QE)/100 g DM. Aliquots of 0.5 mL of each sample were mixed with 1.5 mL of methanol, 0.1 mL of 10% aluminum chloride (AlCl_3_) solution, 0.1 mL of 1 mol/L sodium hydroxide solution (NaOH) and 2.8 mL of distilled water. The resulting mixtures were vigorously mixed and incubated for 30 min in obscurity. The absorbance of the reaction mixture was measured at 430 nm with a UV/visible spectrophotometer. Samples were analyzed in triplicates. The calibration curve was prepared by quercetin solutions at concentrations of 0, 50, 100, 150, and 200 mg/L.

### Determination of the antioxidant activity

The antioxidant activity was investigated using the stable-free radical DPPH assay, according to the method described by Brand-Williams et al. (1995) with modifications. Different dilutions of the methanolic extracts were prepared for each ripening stage. An aliquot of 1 mL of a diluted sample was added to 2 mL of a 0.1 mmol/L DPPH methanol solution and immediately vortexed. The mixture was then incubated at room temperature in the dark for 90 min. Absorbance of the mixture was measured at 517 nm by an UV-visible spectrophotometer. Methanol was used to zero the spectrophotometer. The scavenging activity was calculated from the calibration curve using 6-hydroxyl-2,5,7,8-tertramethylchrome-2-carboxylic acid (Trolox) as a positive standard at the concentrations 1, 1.5, 2, 3, and 4 mmol/L and expressed as a*μ*mol trolox equivalent antioxidant capacity (TEAC)/100 g DM.

### Statistical analysis

The results were expressed as the mean value ± standard deviation of triplicate determinations. Duncan's test was carried out to determine statistical significance between the means. Pearson's correlation coefficient between the antioxidant activity, total phenolics, and flavonoids content of date palm fruit extracts were established. Differences were considered statistically significant at the*P* ≤ 0.05 level. Data were processed using SPSS statistics 17.0 software (SPSS Inc., Chicago, IL).

## Results and Discussion

### Total phenolics

The total polyphenols assessed using Folin–ciocalteu phenol's reagent showed that the*Blah* stage possessed significantly higher amount of phenolics than the fully mature*Tamr* stage regardless the cultivar with mean values of 728.5 and 558.9 mg GAE/100 g DM, respectively (Table2). At the cultivar level, total phenolics ranged from 632.2 to 853.3 mg GAE/100 g DM and 405.5–661.1 mg GAE/100 g DM for*Blah* and*Tamr* stages, respectively. The*Blah* stage of Bou seker cultivar contained significantly high amounts of phenolics (853.3 mg GAE/100 g DM) compared to the others (*P* < 0.05). Estimates of total phenolics in dates using the colorimetric Folin–Ciocalteu method varies greatly according to variety (Al-Farsi et al. 2005) and phenolic standards used as well as the units used to express the data (fresh or dried matter). Our data are in agreement with previous studies indicating that phenolic content in fruit of date palm decrease as ripening progressed (Awad et al. 2011; Eid et al. 2013; Hammouda et al. 2013). Thus, Awad et al. (2011) founded total phenolics concentrations in the range of 50–210 and 10–80 mg/100 g fresh weight (FW) at the*Biser* and the*Rutab* stage, respectively, in fruits of five date palm cultivars grown in Saudi Arabia. In addition, Hammouda et al. (2013) showed that the amount of phenolic compounds decreased linearly, with a 25% loss from the Khalal to the Tamar stage in the flesh of two date palm cultivar from Tunisia. However, Allaith (2008) founded an average of phenolics in two Bahraini date cultivars (Hallaw and Khalas), that decreases from*Biser* to*Rutab* stage from 232 and 189 to 90.3 and 128.4 mg GAE/100 g FW, respectively, and then increases in the*Tamar* stage to 342 and 276 mg GAE/100 g FW, respectively. The degree of ripeness considerably affects the concentrations and proportions of the various polyphenols in edible plants. In general, it has been observed that phenolic acid concentrations decrease during ripening (Manach et al. 2004). For instance, Aziz et al. (1976) observed a general decline in total phenolic content in the pulp of banana fruit during ripening. Similar findings have been also reported by Venkatesan and Tamelmani (2010) in the fruit of mango (*Mangifera indica* L. var. Neelum). On the other hand, it has been observed that the loss of astringency over the course of ripening is associated with the loss of phenolic content (Selvaraj and Kumar 1989).

**Table 2:** Means ± standard deviations of total phenolics and flavonoids content of six date palm cultivars at the*Blah* and*Tamr* stages.

Cultivar	Total phenolics (mg GAE/100 g DM)		Flavonoids content (mg QE/100 g DM)	
	*Blah* (*Khalal*)	*Tamr*	*Blah* (*Khalal*)	*Tamr*
Ahmar dli	803.3 ± 8.8b	610 ± 4.4c	127.7 ± 1.87b	112.5 ± 0.83a
Ahmar denga	604.4 ± 3.0f	527 ± 7.1d	106.8 ± 1.25d	48.9 ± 0.6c
Bou seker	853.3 ± 5.2a	467.7 ± 7.7e	134.3 ± 0.83a	46.1 ± 1.98c
Tenterguel	632.2 ± 6.5e	405.5 ± 2.2f	97.9 ± 0.84d	39.5 ± 1.5d
Lemdina	691.1 ± 6.6d	622.2 ± 6.6b	136.4 ± 1.2a	81.2 ± 0.55b
Tijib	786.6 ± 5.5c	661.1 ± 3.1a	114.3 ± 1.25c	75.2 ± 1.04b
Average	728.5 ± 95.8	558.9 ± 96.9	119.6 ± 14.7	67.3 ± 26.1

Values appended by a different letter are significantly different at the 0.05 probability level. GAE, gallic acid equivalent; DM, dry matter; QE, Quercetin equivalent.

### Flavonoids content

Flavonoids content in fruit of tested date palm cultivars are shown in Table2. Significant variation in flavonoids concentration existed within the same cultivar at*Blah* and*Tamr* stages as well as between different cultivars. The cultivar Tenterguel showed the lowest flavonoid content in both development stages,*Blah* and*Tamr*, with 97.9 and 39.5 mg querectin equivalents (QE) per 100 g DM, respectively. The cultivar that exhibited the highest flavonoids in the*Blah* stage was Lemdina with 136.4 mg QE/100 g DM. The overall averages of flavonoids content in the*Blah* and*Tamr stages* were 119.6 and 67.3 mg QE/100 g DM, respectively. The percent decrease in flavonoids content ranged from 11.9% to 65.6% in Ahmar dli and Bou seker dates, respectively, and the overall average of flavonoid decrease was 43.7% (data not shown). Eid et al. (2013) and Hammouda et al. (2013) using high-performance liquid chromatography (HPLC) and reverse phase-HPLC (RP-HPLC) reported the same trends when they compared the flavonoid content during the development stages in date varieties from Saudi Arabia and Tunisia, respectively. The decline in flavonoid content over the course of maturation was also observed in the oil palm fresh fruit bunches (Hazir et al. 2012).

### Antioxidant activity

The antioxidant activity of fruit extracts was assessed using the DPPH assay. The study revealed that cultivars at the*Blah* stage exhibited the highest antioxidant activity with an average of 107.5 *μ*mol TEAC/100 g DM and ranging from a low value of 90.5 in Tenterguel cultivar to a high value of 129.3 *μ*mol TEAC/100 g DM in Bou seker cultivar (Table3). At the*Tamr* stage, cultivars showed an antioxidant activity that ranged between 75.6 and 99.3 *μ*mol TEAC/100 g DM with a mean of 91.2 *μ*mol TEAC/100 g DM. The lowest TEAC value was found in Tenterguel cultivar (75.6 *μ*mol/100 g DM) and the highest value was scored in Tijib cultivar (99.3 *μ*mol/100 g DM). Statistical evaluation of the TEAC values revealed significant variations between date palm cultivars and to some extent between the ripening stage. Indeed, at the*Blah* stage, each cultivar forms a distinct group while, at the*Tamr* stage, only three groups were observed. On the other hand, antioxidant activity declines among the various cultivars between the maturation stages,*Blah* and*Tamr*. The percentage lost of the antioxidant activity between the two stages ranged from 0.4% in the Ahmar denga variety to 39.1% in Bou seker, with an average of 15.1%. It is noticeable that cultivar Bou seker which initially showed the greatest level of antioxidant activity at the*Blah* stage, had the greatest decline (39.1%) in this activity in the*Tamr* stage. The antioxidant activity of dates was reported by several authors (Allaith 2008; Chaira et al. 2009; Saafi et al. 2009; Awad et al. 2011; Hammouda et al. 2013). Although different assays has been used in the assessment of their antioxidant activity such as DPPH, ferric-reducing antioxidant potential (FRAP), 2,2′-azino-bis (3-ethylbenzthiazoline)-6-sulfonic acid (ABTS), they all concluded to the powerful antioxidant activity of date fruit at both ripening stages. For instance, Saafi et al. (2009) using the DPPH assay reported antioxidant activity values that ranged from 866.82 to 1148 *μ*mol trolox equivalents/100 g FW for the*Tamr* stage in Deglet nour and Khouet Kenta date palm fruit, respectively. Mansouri et al. (2005), using the same assay to estimate the antioxidant activity expressed as the mass ratio (*μ*g sample/*μ*g DPPH) in seven different ripe date palm fruits from Algeria, including Deglet nour, founded an antiradical efficiency that ranged from 0.08 to 0.22.

**Table 3:** Means ± standard deviations of antioxidant capacity of six date cultivars at the*Blah* and*Tamr* stages.

Cultivar	Antioxidant activity (*μ*mol TEAC/100 g DM)		% lost activity
	*Blah* (*Khalal*)	*Tamr*	
Ahmar dli	108.4 ± 0.69a	98.9 ± 1.03a	8.7
Ahmar denga	99.1 ± 1.2b	98.7 ± 1.03a	0.4
Bou seker	129.3 ± 0.69c	78.7 ± 0.86b	39.1
Tenterguel	90.5 ± 0.86d	75.6 ± 1.03b	16.4
Lemdina	103.4 ± 1.03e	95.8 ± 0.6c	7.3
Tijib	114.3 ± 1.2f	99.3 ± 0.51a	13.1
Average	107.5 ± 12.6	91.2 ± 10.3	15.1

Values appended by a different letter are significantly different at the 0.05 probability level. TEAC, trolox equivalent antioxidant capacity; DM, dry matter.

### Correlation coefficients and regression analysis

Correlation coefficients between the antioxidant activity value and the total phenolics, flavonoids content, and the stage of ripening are presented in Table4. At*Tamr* and*Blah* stages, correlations were highly significant between antioxidant activity values and total phenolics (*r* = 0.92 and 0.87, respectively). Flavonoids content also showed a significant but a lower correlations with antioxidant activity at the*Tamr* and*Blah* stages (*r* = 0.67 and 0.66, respectively). Regression analysis of antioxidant activity of*Blah* and*Tamr* dates revealed significant positive relationships between TEAC values and total phenolics (*r*^2^ = 0.76 and 0.85, respectively). However, weak but significant relationships between flavonoids content and TEAC in both ripening stages were noted (data not shown). Similar finding were reported by Amoros et al. (2009) who founded a high correlation between phenolic content and hydrophilic total antioxidant activity in the*Khalal* stage of seven date palms from the Elche grove in Spain. In addition, Mansouri et al. (2005) reported a high correlation between phenolic content and the antiradical efficiencies of seven Algerian ripe date palm fruits (*r*^2^ = 0.975).

**Table 4:** Pearon's correlation coefficient between TEAC values and different variables in fruit of six date palm cultivars.

Variable	*Blah*	*Tamr*
Total phenolics	0.87 (<0.0001)	0.92 (<0.0001)
Flavonoids content	0.66 (0.0026)	0.67 (0.002)

*P*-values are given in parentheses. TEAC, trolox equivalent antioxidant capacity.

## Conclusion

Mauritanian date fruits considered in this study could serve as a good source of natural antioxidant and it would be desirable to consume them at the Blah stage in order to achieve the maxima nutritional and functional properties with benefits to human health. Researchers are in progress to determine the spectrum of multiple phenolics and flavonoids representatives.
